# Natural Language Processing of Clinical Notes to Identify Mental Illness and Substance Use Among People Living with HIV: Retrospective Cohort Study

**DOI:** 10.2196/23456

**Published:** 2021-03-10

**Authors:** Jessica P Ridgway, Arno Uvin, Jessica Schmitt, Tomasz Oliwa, Ellen Almirol, Samantha Devlin, John Schneider

**Affiliations:** 1 Department of Medicine University of Chicago Chicago, IL United States; 2 Center for Research Informatics University of Chicago Chicago, IL United States

**Keywords:** natural language processing, HIV, substance use, mental illness, electronic medical records

## Abstract

**Background:**

Mental illness and substance use are prevalent among people living with HIV and often lead to poor health outcomes. Electronic medical record (EMR) data are increasingly being utilized for HIV-related clinical research and care, but mental illness and substance use are often underdocumented in structured EMR fields. Natural language processing (NLP) of unstructured text of clinical notes in the EMR may more accurately identify mental illness and substance use among people living with HIV than structured EMR fields alone.

**Objective:**

The aim of this study was to utilize NLP of clinical notes to detect mental illness and substance use among people living with HIV and to determine how often these factors are documented in structured EMR fields.

**Methods:**

We collected both structured EMR data (diagnosis codes, social history, Problem List) as well as the unstructured text of clinical HIV care notes for adults living with HIV. We developed NLP algorithms to identify words and phrases associated with mental illness and substance use in the clinical notes. The algorithms were validated based on chart review. We compared numbers of patients with documentation of mental illness or substance use identified by structured EMR fields with those identified by the NLP algorithms.

**Results:**

The NLP algorithm for detecting mental illness had a positive predictive value (PPV) of 98% and a negative predictive value (NPV) of 98%. The NLP algorithm for detecting substance use had a PPV of 92% and an NPV of 98%. The NLP algorithm for mental illness identified 54.0% (420/778) of patients as having documentation of mental illness in the text of clinical notes. Among the patients with mental illness detected by NLP, 58.6% (246/420) had documentation of mental illness in at least one structured EMR field. Sixty-three patients had documentation of mental illness in structured EMR fields that was not detected by NLP of clinical notes. The NLP algorithm for substance use detected substance use in the text of clinical notes in 18.1% (141/778) of patients. Among patients with substance use detected by NLP, 73.8% (104/141) had documentation of substance use in at least one structured EMR field. Seventy-six patients had documentation of substance use in structured EMR fields that was not detected by NLP of clinical notes.

**Conclusions:**

Among patients in an urban HIV care clinic, NLP of clinical notes identified high rates of mental illness and substance use that were often not documented in structured EMR fields. This finding has important implications for epidemiologic research and clinical care for people living with HIV.

## Introduction

Behavioral health disorders are highly prevalent among people living with HIV [1,2], who have a 2 to 4-fold higher risk of depression than the general population, with prevalence rates ranging from 24% to 63% [3-9]. A recent study among over 10,000 people living with HIV at seven HIV care sites across the United States found the prevalence of substance use disorder to be 48%, with 20% of patients having polysubstance use disorder [10]. This is higher than the rate of the general US population, in which the prevalence of substance use disorder is 7.7% [11].

In addition to being common among people living with HIV, mental illness and substance use often lead to poor health outcomes for this population. People living with HIV who have mental illness and substance use disorder have lower rates of engagement in HIV care and are less likely to adhere to antiretroviral therapy than those without behavioral health disorders [12-18]. Depression has been independently associated with mortality among several large cohorts of people living with HIV [12,19-21]. Besides poor individual health outcomes, people living with HIV with mental illness or substance use disorder are more likely to transmit HIV to others, because behavioral health disorders are associated with elevated HIV viral loads and behaviors that increase the risk of HIV transmission [22-24]. Many people living with HIV have co-occurring mental health disorders and substance use disorders, further exacerbating these adverse health outcomes [5,14].

To improve understanding of mental illness and substance use among people living with HIV, electronic medical record (EMR)-based behavioral health data are increasingly being utilized in HIV-related clinical research and medical care [25-27]. For example, Tolson et al [25] used an electronic reporting tool within the EMR to identify people living with HIV with substance use disorders to determine the association of substance use with hospitalization and virologic suppression. Other researchers used electronic billing codes to identify risk factors for suicidal ideation among people living with HIV [27]. However, mental illness and substance use are often underdocumented in structured EMR fields (eg, diagnosis codes, Problem List) [26,28,29], potentially leading to the exclusion of people living with HIV with behavioral health disorders from these studies if only discrete EMR data are used.

Natural language processing (NLP) of unstructured text of clinical notes in the EMR may identify behavioral health disorders beyond those identified using structured EMR fields alone [30,31]. Afshar et al [31] used NLP of clinical notes to identify patients with alcohol misuse, demonstrating greater accuracy than EMR-based billing codes; however, this study was performed among hospitalized trauma patients rather than with outpatients living with HIV. Oliwa et al [32] used NLP of clinical notes to identify phrases associated with improved engagement in HIV care. Their study identified NLP phrases related to substance use and mental health among people living with HIV, but they did not compare their findings with documentation in structured EMR fields.

To fill these gaps, the aim of this study was to utilize NLP of clinical notes to detect mental illness and substance use among people living with HIV, and to determine how often these factors were documented in structured EMR fields.

## Methods

We performed a retrospective cohort study among people living with HIV at the University of Chicago Medicine (UCM) in Chicago, Illinois. Participants were included in the study if they were HIV-positive, 18 years of age or older, and attended at least one outpatient HIV care encounter at UCM between May 1, 2011 and May 30, 2016. This study was approved by the University of Chicago Institutional Review Board.

For eligible participants, we collected both structured EMR data as well as the unstructured text of clinical HIV care notes during the study time period. Structured EMR data collected included demographics, diagnosis codes (International Classification of Disease [ICD]-9 and ICD-10), Problem List (a list of physician-assigned diagnoses in the EMR), and social history. Unstructured data included the text of notes written by physicians, advanced practice providers, nurses, and social workers in the Department of Infectious Diseases. Data were extracted from the University of Chicago Clinical Research Data Warehouse, which stores data from the EMR (EPIC, Verona, WI) as well as administrative databases.

To develop the NLP algorithms for detecting mental illness and substance use, subject matter experts (physicians at the Department of Infectious Diseases and HIV care social workers) defined potential indicative words and crafted regular expressions to search for these key words and phrases related to mental illness and substance use (see Textbox 1). NegEx with augmented negation terms was applied to the key words and phrases found in clinical notes [33]. Those that were identified as negated occurrences by NegEx were excluded for the subsequent NLP steps. The Lucene Porter stemmer was used as a stemming algorithm to provide matching generalization between the tokens and the words/phrases from Textbox 1 [34]. Stanford CoreNLP with additional domain-specific split patterns was employed as a tokenizer and sentence splitter to provide the NegEx input sentences [35].

**Words and phrases detected by natural language processing algorithms.**

- Words/phrases for mental illness
  Depression, Depressed, Bipolar, Anxiety, Panic, Psychiatry, Schizophrenia, Bipolar, Psychosis, Care2Prevent (mental health program), Anxious, Therapist (excluding physical therapist), Behavioral health, C2P, Psychotic
*Note: Stemmed forms, regular expression word boundaries, and a negative lookbehind in the case of “therapist” are excluded from this list for readability purposes.*
- Words/phrases for substance abuse
  IVDU (intravenous drug user), Cocaine, Heroin, Crack, Alcohol abuse, AA (Alcoholics Anonymous) meeting, Haymarket (drug treatment program), NA (Narcotics Anonymous) meeting, Drug treatment program

Textboxes 2-4 list the diagnosis codes and Problem List phrases used to identify mental illness and substance use. Structured data from the Social History EMR section was considered to identify substance use if there was documentation of any illegal drug use (with the exception of marijuana) or if there was specific documentation of abuse of substances, including both legal and illegal substances.

To validate the NLP algorithm for mental illness, a random sample of 100 clinical notes flagged as positive for mental illness and 100 clinical notes not flagged for mental illness were manually reviewed to determine if the note documented that the patient had a mental illness. Two reviewers examined each note, and any discrepancies were resolved based on discussion and mutual agreement between reviewers. Using the determination from the manual chart review as the gold standard, we calculated the positive predictive value (PPV) of the algorithm (ie, the number of notes in which mental illness was present based on chart review divided by the number of reviewed notes that were flagged as positive for mental illness). We also calculated the negative predictive value (NPV) of the algorithm (ie, the number of notes in which mental illness was not present based on chart review divided by the number of reviewed notes not flagged by the mental illness algorithm). Similarly, to validate the NLP algorithm for substance use, a random sample of 100 clinical notes in which the algorithm detected substance use and 100 clinical notes where substance use was not detected were manually reviewed. Subsequently, the PPV and NPV for the substance use algorithm were also calculated.

We compared numbers of patients with mental illness or substance use identified by structured EMR fields with those identified by the NLP algorithms.

**International Classification of Diseases (ICD) diagnosis codes used to identify mental illness.**

- ICD-9 codes
  291.9, 293.81, 293.82, 293.83, 293.84, 294.9, 295.3, 295.31, 295.32, 295.33, 295.34, 295.35, 295.42, 295.44, 295.6, 295.7, 295.71, 295.72, 295.75, 295.8, 295.9, 295.92, 296, 296.01, 296.02, 296.1, 296.15, 296.2, 296.21, 296.22, 296.23, 296.24, 296.25, 296.26, 296.3, 296.31, 296.32, 296.33, 296.34, 296.35, 296.36, 296.4, 296.41, 296.42, 296.44, 296.5, 296.51, 296.52, 296.53, 296.54, 296.55, 296.6, 296.64, 296.7, 296.8, 296.9, 297.1, 297.9, 298.9, 300, 300.01, 300.21, 300.3, 300.4, 300.81, 301.7, 301.82, 301.83, 301.9, 309, 309.24, 309.28, 309.3, 309.4, 309.81, 310.8, 311, 312.81, 312.82, 313.81, 314, 314.01, 648.41, 648.44, E950.0, E950.2, E950.3, E950.4, E950.9, E953.0, V11.0, V40.0, V40.9
- ICD-10 codes
  F0630, F09, F203, F2089, F209, F23, F250, F251, F259, F3110, F3111, F312, F3130, F3132, F3170, F3181, F319, F321, F323, F329, F330, F331, F332, F333, F3340, F3341, F3342, F339, F4001, F4010, F411, F419, F4323, F458, F509, F603, F900, F901, F913, F919, Z915

**International Classification of Diseases (ICD) diagnosis codes used to identify substance use.**

- ICD-9 codes
  291, 291.2, 291.3, 291.81, 291.9, 304, 304.01, 304.02, 304.2, 304.22, 304.23, 304.3, 304.31, 304.7, 304.71, 304.72, 304.8, 304.83, 305, 305.01, 305.02, 305.03, 305.2, 305.21, 305.22, 305.23, 305.4, 305.5, 305.51, 305.52, 305.53, 305.6, 305.61, 305.62, 305.63, 305.7, 305.91, 305.93, 425.5, 535.3, 571, 571.2, 648.33, 965.01, 970.81, E850.0, E850.1, E850.2, E854.8, E860.0, E860.9, E935.0
- ICD-10 codes
  F1010, F10120, F10129, F10188, F1020, F1021, F10239, F1029, F1099, F1110, F1120, F1121, F1123, F1190, F11959, F1210, F1220, F1290, F12929, F1410, F14129, F1414, F14188, F1420, F1421, F14259, F1490, F14929, F1494, F1510, F1520, I426, K7031, K852, K860, O99313

**Problem List phrases.**

- Mental illness
  ADD (attention deficit disorder); ADHD (attention deficit hyperactivity disorder); ADHD (attention deficit hyperactivity disorder), inattentive type; ADHD, predominantly inattentive type; Adjustment disorder with depressed mood; Adjustment disorder with mixed anxiety and depressed mood; Agoraphobia with panic disorder; Anxiety; Anxiety and depression; Anxiety disorder; Anxiety disorder in conditions classified elsewhere; Anxiety state, unspecified; Anxiety, mild; Attention deficit disorder without mention of hyperactivity; Bipolar 1 disorder; Bipolar 2 disorder; Bipolar affective; Bipolar affective disorder; Bipolar affective disorder, currently depressed, moderate; Bipolar depression; Bipolar disorder; Bipolar disorder, currently in remission of unspecified degree, most recent episode type unspecified; Bipolar disorder, unspecified; Bipolar I disorder, most recent episode depressed; Bipolar I disorder, most recent episode depressed, severe with psychosis; Bipolar I disorder, most recent episode manic; Bipolar I disorder, most recent episode manic, mild; Borderline personality disorder; Bulimia nervosa; Depressed mood; Depression; Depression (disease); Depression with anxiety; Depression, major; Depression, major, recurrent, severe with psychosis; Depression, recurrent; Depressive disorder; Depressive disorder, not elsewhere classified; Depressive episode; H/O attempted suicide; History of depression; Major depression; Major depression, recurrent; Major depression, recurrent, chronic; Major depressive disorder; Major depressive disorder, recurrent episode, in full remission; Major depressive disorder, recurrent episode, in partial or unspecified remission; Major depressive disorder, recurrent episode, mild; Major depressive disorder, recurrent episode, moderate; Major depressive disorder, recurrent episode, severe, without mention of psychotic behavior; Major depressive disorder, recurrent episode, unspecified; Major depressive disorder, severe; Major depressive disorder, single episode in full remission; Major depressive disorder, single episode, moderate; Manic depression; MDD (major depressive disorder), recurrent episode; MDD, recurrent episode, moderate; Mechanical complication of other vascular device, implant, and graft; Mood disorder; Mood disorder due to known physiological condition; Mood disorder in conditions classified elsewhere; Panic attack; Panic attacks; Panic disorder without agoraphobia; Paranoia (psychosis); Paranoid schizophrenia, chronic condition; Paranoid schizophrenia, chronic condition with acute exacerbation; Paranoid schizophrenia, unspecified condition; Postpartum depression; Posttraumatic stress; Posttraumatic stress disorder; Psychiatric illness; Psychiatric pseudoseizure; Psychosis; Psychosis, organic; Psychotic disorder with delusions in conditions classified elsewhere; PTSD (posttraumatic stress disorder); Schizoaffective disorder; Schizoaffective disorder, unspecified condition; Schizophrenia; Schizophrenia, disorganized, chronic with acute exacerbation; Schizophrenia, paranoid type; Schizophrenia, unspecified type; Suicidal ideation; Suicide attempt by drug ingestion; Suicide ideation; Unspecified schizophrenia, unspecified condition
- Substance use disorders
  Addiction, marijuana; Alcohol abuse; Alcohol abuse, continuous drinking behavior; Alcohol abuse, daily use; Alcohol abuse, episodic; Alcohol dependence; Alcohol dependence in remission; Alcohol dependence with acute alcoholic intoxication; Alcohol use; Alcohol withdrawal; Alcoholic cirrhosis of liver; Alcoholism with alcohol dependence; Cannabis use disorder, mild, abuse; Cocaine abuse; Cocaine abuse, in remission; Cocaine addiction; Cocaine dependence, continuous; Cocaine substance abuse; Cocaine use; Cocaine withdrawal; Dementia associated with alcoholism; ETOH abuse; Excessive blood level of alcohol; H/O alcohol abuse; H/O drug abuse; H/O substance abuse; Habitual alcohol use; History of alcohol abuse; History of alcohol use; History of cocaine abuse; History of cocaine use; History of drug abuse; History of heroin abuse; History of opioid abuse; Hx of cocaine abuse; IV (intravenous) drug abuse; IVDU (intravenous drug user); Marijuana abuse; Methadone dependence; Methadone use; Methamphetamine abuse; Opioid abuse, unspecified; Pancreatitis, alcoholic, acute; Polysubstance abuse; Psychoactive substance-induced organic mood disorder

## Results

During the study period, 778 people living with HIV attended at least one HIV care appointment (Table 1). A total of 13,905 clinical notes were included, with a mean of 13 notes per patient (range 1-109). Based on manual review of clinical notes as described above, the NLP algorithm for detecting mental illness had a PPV of 98% and an NPV of 98%. The NLP algorithm for detecting substance use had a PPV of 92% and an NPV of 98%.

**Figure 1:**
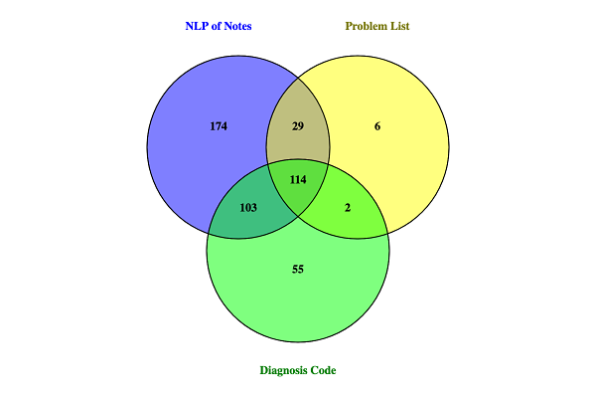
Electronic medical record documentation of mental Illness among people living with HIV. NLP: natural language processing.

The NLP algorithm for mental illness identified 54.0% (420/778) of patients as having documentation of mental illness in the text of clinical notes (Figure 1). With the PPV of the algorithm of 98%, this would suggest that 412 patients truly had mental illness. Among the patients with mental illness detected by NLP, 58.6% (246/420) had documentation of mental illness in at least one structured EMR field (ie, Problem List or diagnosis code), including 34.0% (143/420) with a mental illness listed in the Problem List and 51.7% (217/420) with a diagnosis code related to mental illness. Sixty-three patients had documentation of mental illness in structured EMR fields that was not detected by NLP of clinical notes.

**Table 1:** Demographic characteristics of participants (N=778).

Characteristic		Value
Age (years), mean (SD)		43.1 (13.5)
Female, n (%)		287 (36.9)
**Race/ethnicity, n (%)**		
	Black	620 (79.7)
	White	107 (13.8)
	Latinx	27 (3.5)
	Asian	8 (1.0)
	Other	16 (2.1)
**Insurance, n (%)**		
	Medicaid	272 (35.0)
	Medicare	228 (29.3)
	Private	257 (33.0)
	Other/self-pay	21 (2.7)

**Figure 2:**
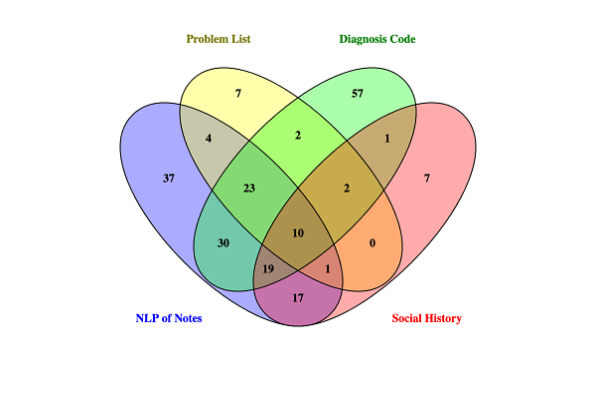
Electronic medical record documentation of substance use among people living with HIV. NLP: natural language processing.

The NLP algorithm for substance use detected substance use in the text of clinical notes in 18.1% (141/778) of participants (Figure 2). Based on the PPV of the algorithm of 92%, it is likely that 130 patients truly had substance use. Among patients with substance use detected by NLP, 73.8% (104/141) had documentation of substance use in at least one structured EMR field, including 27.0% (38/141) with documentation of substance use in the Problem List, 58.2% (82/141) with a diagnosis code related to substance use, and 33.3% (47/141) with substance use documented in the Social History section of the EMR. Seventy-six patients had documentation of substance use in structured EMR fields that was not detected by NLP of clinical notes.

## Discussion

Among patients in an urban HIV care clinic, NLP of clinical notes identified high rates of mental illness and substance use that were often not documented in structured EMR fields. This finding has important implications for clinical care and epidemiologic research among people living with HIV. Namely, relying on structured EMR fields alone to identify people living with HIV with behavioral health disorders may miss a substantial number of patients. Given the high PPV of our algorithms, addition of such NLP algorithms to current tools for identifying behavioral health disorders could augment detection of these disorders among people living with HIV.

To our knowledge, this is the first study to utilize NLP of EMR notes to detect mental illness and substance use among people living with HIV. Other studies have used NLP to detect depression and substance misuse in non-HIV care settings [30,31,36]. Adekkanattu et al [36] used NLP to identify depression from EMR notes among patients prescribed antidepressants, and found that 31% of patients with depression detected by NLP were missing a diagnosis code for depression. Zhou et al [37] similarly used NLP of hospital discharge summaries to identify depression among hospitalized patients, and found that 20% of patients with depression detected by NLP did not have a depression diagnosis code. These rates of discordant documentation are lower than that obtained in this study, in which nearly half of patients with mental illness detected by NLP did not have a diagnosis code for mental illness. This discrepancy may be explained by differences in the patient populations studied. Our patients are from a general HIV clinic, rather than inpatients or outpatients already prescribed antidepressants, populations in which medical providers may be more likely to enter a diagnosis code for mental illness.

Our NLP algorithm for mental illness identified 54% of people living with HIV in our clinical population as having mental illness. This is similar to other studies among people living with HIV, which have shown prevalence rates as high as 63% based on validated depression screening tools (eg, Patient Health Questionnaire-9) [3-9].The NLP algorithm detected substance use in 18% of our clinical population. This rate is within the lower end of what has previously been reported. Hartzler et al [10] found that the prevalence of substance use disorders among people living with HIV at 7 HIV care sites ranged from 21% to 71% based on substance use disorder screening tools. Of note, for both mental illness and substance use, the NLP algorithms failed to flag a substantial number of patients who had mental illness or substance use documented in structured EMR fields, suggesting that NLP algorithms should be used in combination with structured fields rather than as a replacement for structured fields for detecting these characteristics.

As EMR data are increasingly being used for clinical care and research among people living with HIV, extracting accurate behavioral health data from the EMR is essential. EMRs have been used to provide electronic feedback to providers to alert them that patients may have untreated depression [38,39]. Results from NLP of clinical notes could potentially augment such electronic alerts. Recent studies have used structured EMR fields, including documentation of substance use and mental illness, to create predictive models of HIV appointment adherence [12,40]. However, if mental illness and substance use are not adequately documented in structured EMR fields, inclusion of NLP of clinician notes may improve such predictive models by identifying additional risk factors for appointment nonadherence.

Our study has several limitations. We did not review all clinical notes for the presence or absence of behavioral health disorder documentation, and some of the NLP-detected cases may be false positives. Although we adjusted for negation in the text, we may have falsely detected mental illness in some instances where providers wrote in a nonstandard format that patients did *not* have mental illness or where they documented that a family member and not the patient themselves had a behavioral health disorder. In addition, certain phrases (eg, Alcoholics Anonymous meeting) may have detected patients with past substance use disorder rather than active substance use disorder. However, in the review of a random sample of 400 notes, we found a high PPV for the NLP algorithms. The NLP algorithms may have also failed to flag notes that documented behavioral health disorders (ie, false negatives). Moreover, the NLP algorithms do not necessarily detect patients with mental illness or substance use, but only detect documentation in the clinical notes of mental illness or substance use. If providers did not ask patients about these topics or did not document regarding their conversations, then people living with HIV with behavioral health disorders may have been missed by our algorithms. Inclusion of validated behavioral health screening tools within the EMR would likely improve detection of mental illness and substance use. These screening tools were not routinely in place in our clinic at the time of the study, and therefore we were unable to assess how they would have affected the results.

In conclusion, we performed the first study of NLP of unstructured clinical notes for mental illness and substance use among people living with HIV. Although these behavioral health disorders were commonly detected by NLP, they were often undocumented in structured fields of the EMR. More research is needed to understand how to best utilize both structured and unstructured EMR data for clinical and epidemiologic research among people living with HIV.

## Abbreviations

EMR: electronic medical record
ICD: International Classification of Diseases
NLP: natural language processing
NPV: negative predictive value
PPV: positive predictive value
UCM: University of Chicago Medicine
